# Impaired B-cell function in *ERCC2* deficiency

**DOI:** 10.3389/fimmu.2024.1423141

**Published:** 2024-07-11

**Authors:** Raphael Rossmanith, Kai Sauerwein, Christoph B. Geier, Alexander Leiss-Piller, Roman F. Stemberger, Svetlana Sharapova, Robert W. Gruber, Helmut Bergler, James W. Verbsky, Krisztian Csomos, Jolan E. Walter, Hermann M. Wolf

**Affiliations:** ^1^ Immunology Outpatient Clinic, Vienna, Austria; ^2^ Doctoral School Molecular Biology and Biochemistry, Institute of Molecular Biosciences, University of Graz, Graz, Austria; ^3^ Institute for Medical-Chemical Laboratory Diagnostics, Mistelbach-Gänserndorf State Clinic, Mistelbach, Austria; ^4^ Department for Biomedical Research, Center of Experimental Medicine, Danube University Krems, Krems an der Donau, Austria; ^5^ Institute for Immunodeficiency, Center for Chronic Immunodeficiency, Medical Center University of Freiburg Faculty of Medicine, University of Freiburg, Freiburg, Germany; ^6^ Department of Rheumatology and Clinical Immunology, Center for Chronic Immunodeficiency, Medical Center University of Freiburg, Faculty of Medicine, University of Freiburg, Freiburg, Germany; ^7^ Research Department, Belarusian Research Center for Pediatric Oncology, Hematology and Immunology, Minsk, Belarus; ^8^ Department of Dermatology, Venereology and Allergy, Medical University of Innsbruck, Innsbruck, Austria; ^9^ Departments of Surgery, Medical College of Wisconsin, Milwaukee, WI, United States; ^10^ Division of Allergy and Immunology, Department of Pediatrics, Morsani College of Medicine, University of South Florida, Tampa, FL, United States; ^11^ Division of Allergy/Immunology, Department of Pediatrics, Johns Hopkins All Children’s Hospital, St. Petersburg, FL, United States; ^12^ Faculty of Medicine, Sigmund Freud University, Vienna, Austria

**Keywords:** trichothiodystrophy, nucleotide excision repair, DNA repair deficiency, primary immunodeficiency, *ERCC2*, XPD, antibody deficiency, B-cell activation

## Abstract

**Background:**

Trichothiodystrophy-1 (TTD1) is an autosomal-recessive disease and caused by mutations in *ERCC2*, a gene coding for a subunit of the TFIIH transcription and nucleotide-excision repair (NER) factor. In almost half of these patients infectious susceptibility has been reported but the underlying molecular mechanism leading to immunodeficiency is largely unknown.

**Objective:**

The aim of this study was to perform extended molecular and immunological phenotyping in patients suffering from TTD1.

**Methods:**

Cellular immune phenotype was investigated using multicolor flow cytometry. DNA repair efficiency was evaluated in UV-irradiation assays. Furthermore, early BCR activation events and proliferation of TTD1 lymphocytes following DNA damage induction was tested. In addition, we performed differential gene expression analysis in peripheral lymphocytes of TTD1 patients.

**Results:**

We investigated three unrelated TTD1 patients who presented with recurrent infections early in life of whom two harbored novel *ERCC2* mutations and the third patient is a carrier of previously described pathogenic *ERCC2* mutations. Hypogammaglobulinemia and decreased antibody responses following vaccination were found. TTD1 B-cells showed accumulation of γ-H2AX levels, decreased proliferation activity and reduced cell viability following UV-irradiation. mRNA sequencing analysis revealed significantly downregulated genes needed for B-cell development and activation. Analysis of B-cell subpopulations showed low numbers of naïve and transitional B-cells in TTD1 patients, indicating abnormal B-cell differentiation *in vivo*.

**Conclusion:**

In summary, our analyses confirmed the pathogenicity of novel *ERCC2* mutations and show that *ERCC2* deficiency is associated with antibody deficiency most likely due to altered B-cell differentiation resulting from impaired BCR-mediated B-cell activation and activation-induced gene transcription.

## 1 Introduction

In humans gene mutations involved in the nucleotide excision repair pathway (NER) (1) are known to result in three main phenotypes: Trichothiodystrophy (TTD), Xeroderma pigmentosum (XP) and Cockayne syndrome (CS) (2–4). TTD1 is a rare autosomal recessive disease which is caused by mutations in the *ERCC2* gene coding for XPD, an ATP-dependent helicase and component of the human transcription initiation factor TFIIH (5, 6). Mutations in *ERCC2* can cause other phenotypes too, such as XP, XP/CS combination or a mixture of XP/TTD with different clinically severity (5, 6). A review of 112 cases by Faghri et al. in 2008 revealed that *ERCC2* mutations represented the most common genetic defect in TTD patients and were present in almost half of the patients who presented with recurrent and/or severe infection. It is remarkable that in one study 13 of 19 deaths among TTD patients were related to infection and the patients died under the age of 10 years (3). Furthermore, a retrospective study showed that among 13 TTD patients presenting with low serum IgG levels 12 harbored *ERCC2* mutations (7). This indicates that *ERCC2* deficiency is associated with impaired adaptive immunity, in particular antibody deficiency. However, the underlying pathomechanism leading to impaired antibody production in *ERCC2* deficient patients is unknown. To our knowledge only two studies have investigated the adaptive immune system of TTD1 patients by examination of T- and dendritic cell functions and revealed CD4 lymphopenia, skewed T-cell receptor (TCR) repertoire as well as impaired dendritic cell activation and maturation (8, 9). Two TTD1 patients are reported to have received immunoglobulin replacement therapy as treatment against their susceptibility to infections (9, 10). Unfortunately, when IVIG therapy was stopped in one patient the patient died from severe infection thus highlighting the importance of immunological investigation and subsequent treatment in patients with *ERCC2* deficiency.

These previous findings prompted us to study B-cell subpopulations and their function in three unrelated TTD1 patients who presented with classical TTD1 features, developed susceptibility to infection in their early childhood and presented with hypogammaglobulinemia and/or decreased antibody response to vaccination. The patients harbored compound heterozygous *ERCC2* variants, two of them new mutations not previously listed in the literature or common mutation databases. The *ERCC2* mutations were accompanied by defective DNA repair and viability of UV-irradiated patient lymphocytes. Furthermore, our results indicate that impaired B-cell activation contributes to the impaired antibody response in *ERCC2* deficient TTD1 patients and is most likely due to transcriptional dysregulation/dysfunction.

## 2 Materials & methods

### 2.1 Patients and controls

#### 2.1.1 Healthy controls

Healthy anonymous blood donors who fulfilled the required health-prerequisite for Austrian blood donation served as a control group. These controls represent a clinically healthy group of adults (≥18 years). For the evaluation of the cellular immune phenotypes of TTD1 patients we furthermore included age-matched controls either measured in our laboratory or taken from the literature (group 1 (n=40): age 9-13 years, group 2 (n=34): age 14-18 years) (11).

#### 2.1.2 TTD1 patients

**Figure 1:**
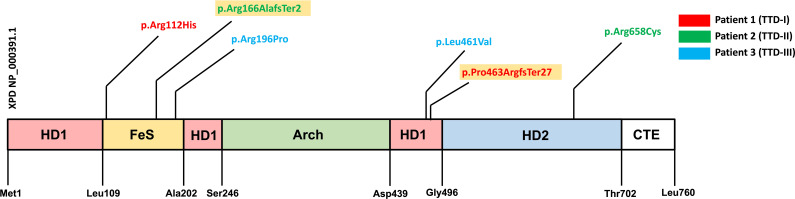
Protein coding variants in the three investigated *ERCC2* deficient patients. Schematic presentation of the XPD protein sequence and mutations harbored by the three TTD1 patients. XPD protein domains are shown [HD1, helicase motor domain 1; FeS, iron sulfur cluster domain; Arch, arch domain; HD2, helicase motor domain 2; CTE, c-terminal extension domain (p44 interacting domain)] and amino acid location. Variants are color coded due to three investigated TTD1 patients. Yellow colored variants indicate truncating mutations.

The genetic evaluation and confirmation of the *ERCC2* mutations in the studied TTD1 patients were carried out using whole-exome sequencing and/or Sanger sequencing. The parents of patients 1 and 2 were confirmed to be heterozygous carriers of the mutations. **Table 1** as well as **Figure 1** provides an overview of the identified mutations. In-silico prediction models (PHRED, REVEL) were queried, and their prediction values regarding the pathogenicity of the known mutations are shown in **Table 1**.

**Table 1:** *ERCC2* variants in investigated TTD1 patients.

Patient	Variant*	cDNA (NM_000400.4)	Protein (NP_000391.1)	dbSNP (rs-ID)	References(PMID)	via CADD CADD PHRED	via dbNSFP CADD PHRED	REVEL rank score
1 (TTD-I)	SNP	c.335G>A	p.Arg112His	rs121913020	7920640; 9758621; 11709541	33	33	0,96725
1 (TTD-I)	Dup	c.1378-7_1387dup	p.Pro463ArgfsTer27	rs2123250560	NA	6.447	NA	NA
2 (TTD-II)	SNP	c.1972C>T	p.Arg658Cys	rs121913021	8571952; 11242112	33	33	0,9721
2 (TTD-II)	Ins	c.494_495insT	p.Arg166AlafsTer2	NA	NA	34	NA	NA
3 (TTD-III)	SNP	c.1381C>G	p.Leu461Val	rs121913016	7849702; 8571952; 9195225; 9758621	34	34	0,90939
3 (TTD-III)	SNP	c.587G>C	p.Arg196Pro	rs753293260	NA	32	32	0,96836

NA, not available; *SNP, single nucleotide polymorphism; Dup, duplication; Ins, insertion.

Patient 1 (TTD-I) is a woman who is 22-years old and suffered from recurrent infections in the first years of life. She harbored compound heterozygous mutations in the *ERCC2* gene, a mutation already described as pathogenic (NCBI dbSNP: rs121913020) (NM_000400.4: c.335G>A, NP_000391.1: p.Arg112His) and furthermore a previously unknown 17 bp duplication (NM_000400.4: c.1378-7_1387dup, NP_000391.1: p.Pro463ArgfsTer27) in the intron 14/22 exon 15/23 boundary. This duplication leads to a frameshift with a premature stop-codon, most likely to a reduced expression of the truncated protein or the loss of functional XPD domains.

Patient 2 (TTD-II) is an 11-year-old boy presenting with a history of recurrent infections. Patient 2 carried the following compound heterozygous *ERCC2* mutations: c.494_495insT (NM_000400.4, NP_000391.1: p.Arg166AlafsTer2), a previously unknown mutation, causing a reading frameshift and premature stop-codon leading to the loss of p.167-760 and an already described pathogenic mutation (NCBI dbSNP: rs121913021) (NM_000400.4: c.1972C>T, NP_000391.1: p.Arg658Cys) on the other allele.

Patient 3 (TTD-III) is a woman who is 18 years old and suffered from TTD, XP and immunodeficiency in her childhood. This patient was studied by Chang et al. in 2008 who reported T-cell dysfunction and impaired antibody production (12). She carries a previously described (NCBI dbSNP: rs121913016) pathogenic heterozygous *ERCC2* mutation (NM_000400.4: c.1381C>G, NP_000391.1: p.Leu461Val) and furthermore an *ERCC2* gene mutation with currently unknown significance (ClinVar Miner) (NM_000400.4: c.587G>C, NP_000391.1: p.Arg196Pro) but predicted to be deleterious by in silico analysis (https://www.ncbi.nlm.nih.gov/clinvar/variation/17502529). Furthermore, the functional results presented in this study confirmed a previous study (12) by showing that her LCL cells’ UV sensitivity was defective to an extent comparable to that of cells from patients 1 and 2, a cellular defect known to be associated with defective NER due to *ERCC2* deficiency, which together with the clinical presentation indicates a pathogenic effect of the R196P mutation.

### 2.2 Analysis of serum immunoglobulins and antibody response

Serum Ig levels were measured by using nephelometric analysis. Antibody responses following vaccination were determined by commercially available ELISA kits (Vacczyme human anti Tetanus toxoid EIA Kit, Vacczyme human Diphtheria Toxoid EIA Kit and Vacczyme human anti Haemophilus Influenzae EIA Kit (all from Binding Site, Birmingham, United Kingdom)) and antibodies against pneumococcus were assessed using an in-house ELISA, which can be used for the simultaneous detection of 23 pneumococcus capsular polysaccharide serotypes (13).

### 2.3 Lymphocyte subsets

Lymphocyte subpopulations were analyzed in peripheral blood using multicolor flow cytometry analysis. Anticoagulated whole blood samples served as starting material and were used for standard flow cytometry staining procedures. To 100 μl whole blood we added fluorescence labeled anti-human mononuclear antibodies (anti-human CD45 (2D1), anti-human CD19 (HIB19), anti-human CD27 (0323), anti-human IgD (IA6-2), anti-human IgM (SA-DA4) and anti-human CD38 (HIT2)) as recommended by manufacturers prescriptions and incubated for 30 minutes at room temperature in the dark. Then the red blood cells were lysed, and leukocytes fixed by addition of 2 ml 1x BD^®^ FACS™ Lysing Solution (Beckton Dickinson, New Jersey, USA) and incubated for 20 minutes. Thereafter, the cells were washed by centrifugation (400xg, 5 minutes, 8°C) and addition of 2 ml PBS (0.1% NaN_3_) twice. Finally, the cells were resuspended in 100 μl PBS (0.1% NaN_3_). The stained cells were measured and analyzed on BD^®^ FACSVerse™ using FACSuite™ software (Beckton Dickinson, New Jersey, USA). Exclusion of cell-duplets and debris was performed by discrimination per single-cell-gating (FSC-A versus FSC-H). B-cell subpopulations were determined by the following gating strategy: Naïve B-cells CD45^+^ CD19^+^ IgD^+^ CD27^-^, Memory B-cells CD45^+^ CD19^+^ IgD^-^ CD27^+^, Transitional B-cells CD45^+^ CD19^+^ IgD^+^ CD27^-^ CD38^high^ IgM^high^. Age matched controls served as reference for the evaluation of lymphocyte subsets, either measured in our laboratory or taken from the literature (source: Garcia-Prat et al., 2019).

### 2.4 BCR-mediated B-cell activation

PBMCs from patients and controls were isolated from anticoagulated whole blood samples using density gradient centrifugation (Lymphoprep™). BCR stimulation was performed using formaldehyde fixed and heat-inactivated *staphylococcus aureus* cells bearing protein A (SAC) (Pansorbin^®^ by Sigma Aldrich, Missouri, USA) plus recombinant human IL-2 (final dilution/concentration: 1:10,000 SAC; 100 U/ml human IL-2). The cells were stimulated for 24 hours before up-regulated activation markers CD69 and CD86 on naïve and memory B-cells (B-cell subsets were gated as described in 3.3) were stained with monoclonal antibodies (anti-human CD69 (FN50), anti-human CD86 (IT2.2)) and finally measured by flow cytometry. As a control, unstimulated cells were cultured in medium alone (RPMI 1640 Gibco, Penicillin Streptomycin, 10% FBS). Results are displayed as mean fluorescence intensity (MFI).

### 2.5 TH-cell activation

PBMCs from patient 1 and 10 healthy controls were isolated as described above and cultured in medium (RPMI 1640 Gibco, Penicillin Streptomycin, 10% FBS). T-cell stimulation was induced by addition of Phorbol 12-Myristate 13-Acetate (PMA) and Ionomycin (both from Beckton Dickinson, New Jersey, USA) (final concentrations: PMA 25 mg/ml, Ionomycin 1 µg/ml) and an incubation for 4 hours. Then the cells were harvested and prepared for multicolor flow cytometry measurement using corresponding antibodies (anti-human CD69 (FN50), anti-human CD19 (HIB19), anti-human CD5 (UCHT2), anti-human CD45 (2D1)). The up-regulation of the activation marker CD69 on CD5^+^ T-cells (CD45^+^ CD8^-^ CD19^-^) was measured. As a negative control, unstimulated cells were used.

### 2.6 UV-irradiation assays

An UV-irradiation assay for *Epstein-Barr virus* (EBV)-transformed B-cells (LCLs) was developed to evaluate the cellular response and survival within 7 days following UV-irradiation. Therefore, LCLs of eight healthy blood donors and three TTD1 patients were kept in exponential growth conditions before. UV-irradiation (predominant emission energy: 253,7 nm) was performed using a GS Gene Linker^®^ UV Chamber (Bio-Rad Laboratories Inc., California, USA). We selected an UV dose that led to approximately 50% surviving cells in healthy control cells after 24-48 hours of recovery following UV-irradiation. For viability staining the cells were treated with Zombie Violet™ dye dilution, fixed, permeabilized and finally stained for intracellular detection of γ-H2AX (anti-human γ-H2AX (2F3)) and active-caspase-3 (anti-human active-caspase-3 (CPP32)) following standard methodology. γ-H2AX was used as a biomarker for intracellular DNA damage and its repair following UV-irradiation. The higher the levels of γ-H2AX in the nucleus the more DNA damage is present in living cells (14). Samples were measured and analyzed on BD^®^ FACSVerse™ using FACSuite™ software. We isolated peripheral blood mononuclear cells (PBMCs) from three anonymous blood donors and patient 1 to test the sensitivity to UV-irradiation in peripheral T-cells and B-cells. 1x10^6^ cells/ml were transferred to petri dishes and rested overnight before UV-irradiation was performed as described above. After 6-, 24- and 48-hours the cells were stained using anti-human CD3 (UCHT1), anti-human CD4 (SK3) and anti-human CD8 (RPA-T8) antibodies before intracellular staining was performed by addition of anti-human CD20 (intracellular domain) (H1(FB1)) and anti-human γ-H2AX antibodies.

### 2.7 Proliferation assays

To test the proliferation of UV-irradiated LCL cell suspensions containing 1x10^5^ cells/well were incubated in triplicates in 96-well plates. The cells were UV-irradiated with the same dose as used for flow cytometry experiments. Untreated cells served as negative controls. We analyzed the resulting proliferation from the hour 24 up to 48 (2nd day) by addition of thymidine-[methyl-^3^H] and detection of incorporated radioactivity using a Microbeta^®^ Scintillation Counter 1450 (Perkin Elmer Inc., Massachusetts, USA). Detected beta-radiation was calculated in disintegrations per minute (DPM), results are expressed as percentage of untreated control cells.

### 2.8 Whole blood pokeweed mitogen (PWM) stimulation

Heparin-anticoagulated whole blood was diluted 1:20 in medium. 200 μl of the suspension were transferred to 96-well plates followed by addition of 20 μl stimuli. For each concentration of PWM stimulus, we transferred blood suspensions in triplicate. PWM was diluted in medium and added to reach final concentrations (10 mg/ml (1:10), 1 mg/ml (1:100)). The cells were stimulated by addition of PWM stimulus and incubated for 7 days. To measure proliferation activity, 16 hours before the reactions were stopped by freezing 20 μl of thymidine-[methyl-3H] (2 μCi/ml) were added to each well. The preparation and measurement of beta-radiation was performed exactly as previously described (3.7) and detected radiation was calculated as disintegrations per minute (DPM).

### 2.9 Western blot analysis

We used LCL generated from cells of TTD1 patient 1 and patient 2 who are carrier of compound heterozygous null alleles (due to limited access to cellular material patient 3 was not investigated). LCLs were harvested and resuspended in a mixture of lysis buffer. The suspension was incubated on ice and centrifuged. Supernatant was then collected and total protein concentration was determined using the DC Protein Assay (BioRad, California, USA). Lysate supernatant containing 20 µg of total protein was denatured in Laemmli-Buffer (BioRad, California, USA). NuPAGE™ 4 to 12%, Bis-Tris, 1.0–1.5 mm, Mini Protein Gels (Invitrogen, Massachusetts, USA) and NuPAGE™ MOPS SDS Running Buffer (20X) (Invitrogen, Massachusetts, USA) was used for electrophoresis. The gel was placed on Nitrocellulose-membrane and the blotting sandwich assembled according to the manufacturers protocol. Blotting was performed using the “NuPAGE Blot” preset and the NuPage Transfer Buffer (1x) (Invitrogen, California, USA) including 20% Ethanol, antioxidants (Invitrogen, California, USA). Afterwards the membrane was removed, and unspecific binding sites blocked using milk powder in TBS + 0.05% Tween 20. When the blocking solution was removed rabbit anti human ERCC2 (XPD) polyclonal antibody (Sigma, Missouri, USA) at 1 µg/ml was added. Alternatively, we used the rabbit anti human GAPDH antibody (Invitrogen, Massachusetts, USA) at 100 ng/ml as positive control. The primary antibody was incubated overnight. HRP-conjugated antibody served as secondary antibody (BioRad, California, USA). Finally, detection was performed using a chemiluminescent substrate and was detected by a ChemiDoc (BioRad, California, USA) chemiluminescence detector.

### 2.10 Differential gene expression and IGH-chain transcript analysis in PBMCs

mRNA sequencing data was generated from PBMCs of five healthy controls and three TTD1 patients. Total RNA was isolated using Monarch^®^ Total RNA Miniprep Kit (New England Biolabs Inc., Massachusetts). Library preparation, sequencing and bioinformatical analysis as differential mRNA expression was performed by Matthias Hackl, PhD and team (TAmiRNA GmbH, Vienna, Austria). mRNA sequencing data (RPM normalized, p < 0.05) were used for statistical comparison of *IGHD-*, *IGHM-* and *IGHG (* 1–4) gene expression in five HCs and the TTD1 patients. Gene expression of IGH chains was normalized to absolute B-cell counts (*IGHD* and *IGHM* to absolute counts of naïve B-cells, *IGHG* to absolute counts of memory B-cells) detected in peripheral blood samples. For evaluation of total IgG expression *IGHG* subclass (1 to 4) expressions were summarized. Raw and processed data files are available on Gene Expression Omnibus – NCBI (GEO) with accession ID: GSE262217.

### 2.11 Statistical analysis

For statistical evaluation Graphpad Prism 10 software (GraphPad Software, California, USA) was used. Statistical differences between two groups were experimentally confirmed by using the non-parametric two-tailed Mann-Whitney U-test. Statistical significance was indicated as followed: ns = not significant, * p=<0.05, ** p=<0.01, *** p=<0.001, **** p=<0.0001.

## 3 Results

### 3.1 XPD protein levels are decreased in *ERCC2* deficient B-cells

**Figure 2:**
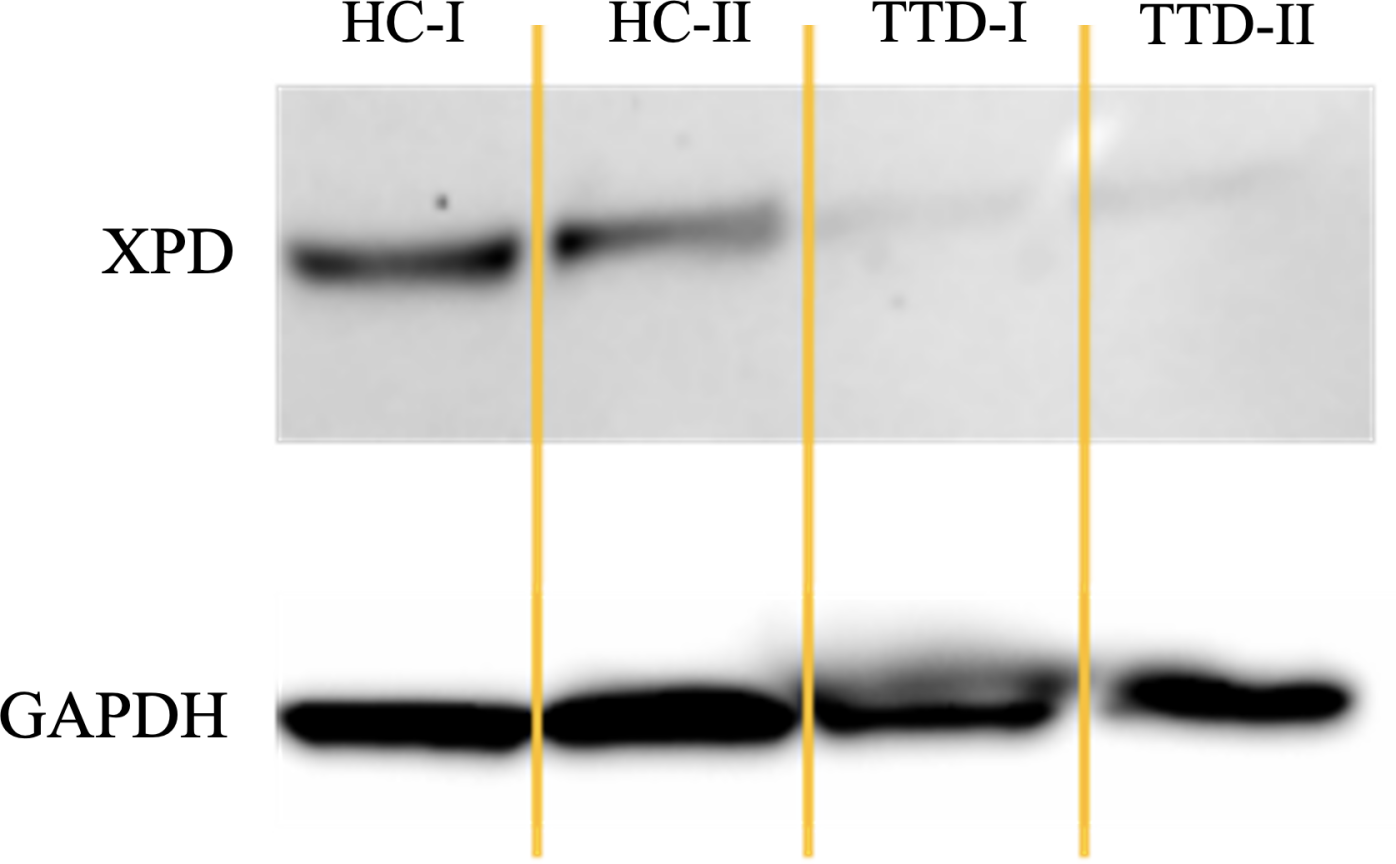
XPD protein expression in TTD1 patients as compared to that of the housekeeping gene GAPDH. LCLs from two healthy controls (HC-I and HC-II) and two patients with trichothiodystrophy-1 (patient 1: TTD-I, patient 2: TTD-II) were harvested, lysed, and examined by Western Blot analysis.

Western blot analysis revealed drastically reduced XPD protein levels in LCLs from TTD1 patients 1 and 2 harboring novel *ERCC2* variants. (**Figure 2**) Unfortunately, expression of XPD protein could not be examined in patient 3 because material from this patient’s LCL cells was not available.

### 3.2 *ERCC2* deficiency leads to UV sensitivity in T- and B-cells

**Figure 3:**
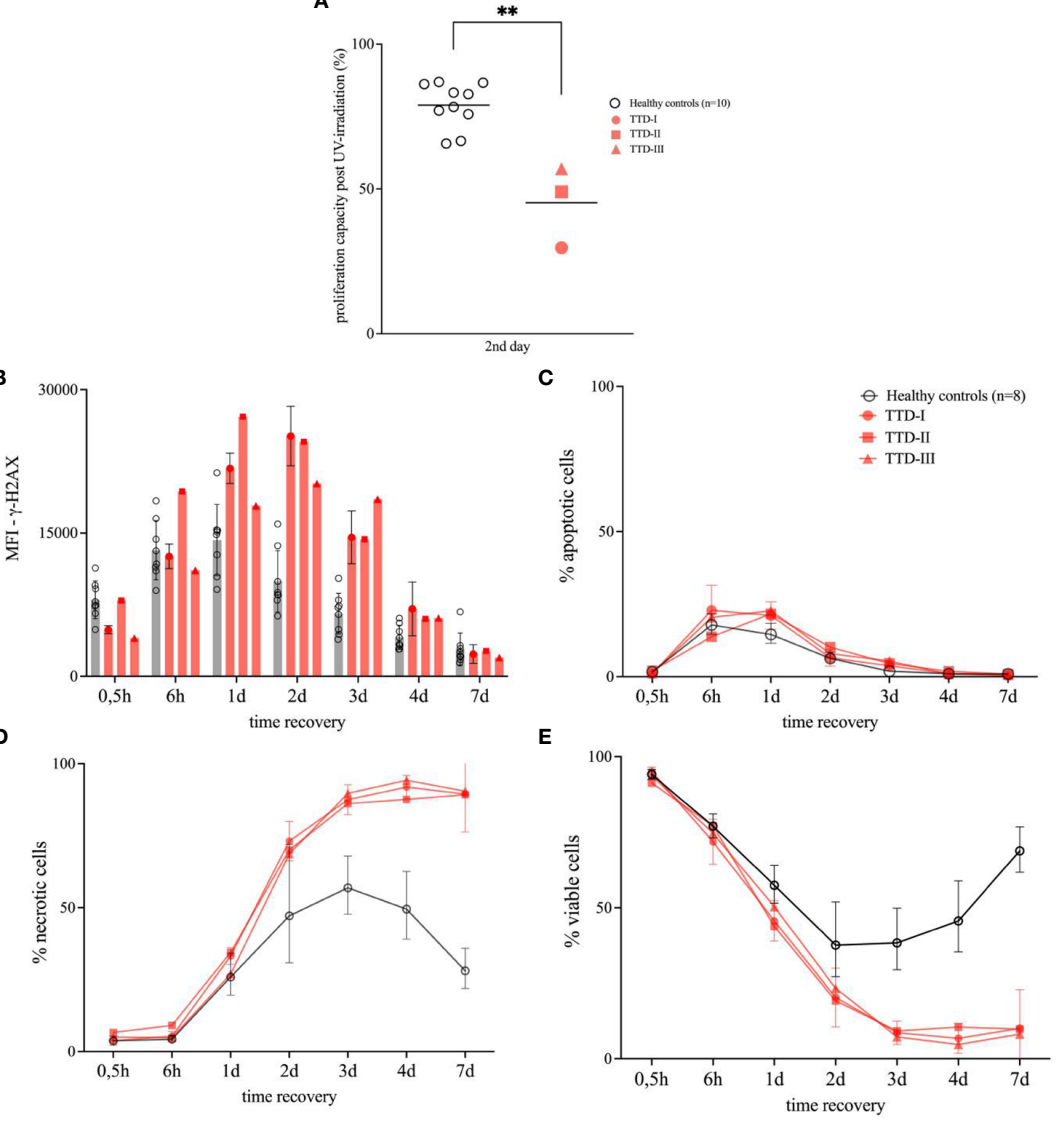
Cellular response to UV-irradiation in lymphoblastoid cell lines (LCLs). LCLs were generated from ≥8 healthy anonymous blood donors (HCs) as well as TTD1 patients (TTD-I, TTD-II and TTD-III) and were used for UV-irradiation experiments. **(A)** Proliferation capacity of UV-irradiated LCLs. Non-irradiated control cells served as reference for calculation of proliferation activity in each sample (proliferation activity measured in disintegrations per minute (DPM)). LCL cultures were UV-irradiated and thymidine-[methyl-3H] was added 24 hours (2nd day) later. Cells were then incubated for 24 hours to investigate proliferation. Relative values represent proliferation capacity following UV-irradiation. Individual values from HCs are shown as rings and line indicates mean. TTD1 patients are shown in red and line indicates mean. (p-value: **p < 0.01, non-parametric Mann-Whitney U test) **(B–E)** Viability following UV-irradiation in LCLs. Intracellular signals of γ-H2AX (**B**, DNA damage), active caspase-3 (**C**, apoptotic cells) and viability dye (**D**, necrotic cells) were evaluated by intracellular multicolor flow-cytometry analysis following UV-irradiation and recovery. Mean fluorescence intensities (MFI) represent γ-H2AX levels. Percentage apoptotic cells represent amount of active-caspase-3 positive cells in the culture and percentage necrotic cells were identified by increased levels of intracellular signals of viability dye. Percentage viable cells **(E)** represent the sum of non-apoptotic and non-necrotic cells in each UV-irradiated cell culture. Non-UV-irradiated cells from each tested individual served as untreated controls (data not shown). Error bars show standard deviations from the mean in HCs. Error bars of TTD1 patient 1 represent the mean of ≥3 performed experiments.

UV-irradiation led to a reduction of the LCL proliferation to 59% in cells from patient 1, 79.5% in cells from patient 2 and 56.58% in patient 3 cells within the first 24 hours post UV-irradiation (data not shown). During the hours 24-48 post UV-irradiation the proliferation decreased further down to 25% in patient 1 cells, 51.45% in patient 2 cells and 57% in patient 3. In comparison the mean proliferation value of the HCs reached 79.95% in the hours 24-48 post UV-irradiation (**Figure 3A**).

**Figure 4:**
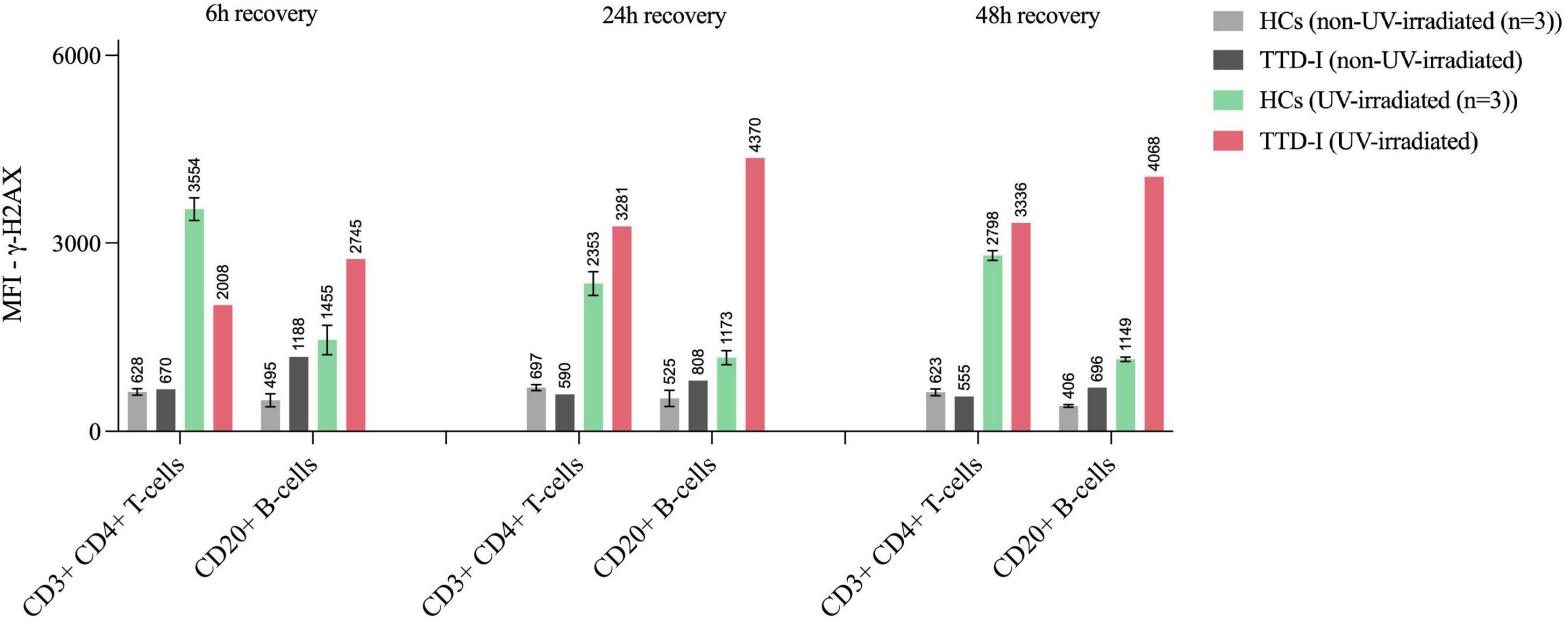
DNA damage response of UV-irradiated lymphocyte subpopulations. Intracellular γ-H2AX intensities following UV-irradiation. CD3^+^CD4^+^ T-cells and CD20^+^ B-cells from 3 healthy controls (HCs) and a TTD1 patient (patient 1). PBMCs were isolated from whole blood via density grade medium and rested overnight. Cells were then UV-irradiated and recovered for 6, 24 and 48 hours. Intracellular staining procedure and multicolor flow cytometry analysis was performed. Mean fluorescence intensities (MFI) represent γ-H2AX levels. Non-irradiated cells served as negative controls (light grey= HCs, dark grey=TTD-I) for UV-irradiated samples (light green= HCs, red= TTD-I). Error bars show the standard deviation from the mean and top numbers represent mean values.

Viable cells represented as non-apoptotic and non-necrotic cells were strongly decreased in TTD1 LCLs from the second day on until the 7^th^ day of recovery (post UV-irradiation) as compared to LCLs from HCs. (**Figure 3E**) The apoptosis following UV-irradiation was not significantly different in healthy controls and TTD1 cells (**Figure 3C**). In contrast, necrotic cell death was strongly increased in LCLs from TTD1 patients as compared to healthy control cells (**Figure 3D**). Furthermore, to confirm DNA repair deficiency in TTD1 lymphocytes γ-H2AX levels were measured. After 2 days of recovery post UV-irradiation γ-H2AX levels were twofold increased in cells from TTD1 patients as compared to the mean of healthy control cells (**Figure 3B**). DNA repair was also investigated in primary CD4^+^ T-cells and CD20^+^ B-cells and the results revealed significantly elevated γ-H2AX intensities in CD4^+^ T-cells and B-cells of patient 1 as compared to cells from three healthy controls. After 24- and 48-hours recovery the cells from patient 1 still showed drastically increased γ-H2AX levels (**Figure 4**).

### 3.3 Impaired antibody response and hypogammaglobulinemia in TTD1 patients

Patient 1 presented with severe hypogammaglobulinemia (IgG 79 mg/dl, IgM 48 mg/dl) as well as drastically reduced IgG antibody response to vaccination (**Table 2B**) at the age of one year so that immunoglobulin replacement therapy was initiated. The inability to produce antibodies was further confirmed during immunoglobulin replacement therapy when patient 1 was repeatedly (seven times) vaccinated against tick-born encephalitis and nevertheless showed ten-fold decreased tick-born encephalitis serum IgG as compared to healthy controls (**Table 2C**; standard human immunoglobulin products have been shown to contain only low levels of TBEV IgG antibodies), thus confirming severe impairment of IgG antibody production. Patient 2 had a history of recurrent infections. He also showed impaired IgG antibody responses to vaccination antigens (**Table 2B**) and borderline low levels of total serum IgM (72 mg/dl) (**Table 2A**). Patient 3 showed decreased total serum IgM levels (74 mg/dl) but evaluation of IgG antibody response was not possible because she refused TBEV vaccination and already received subcutaneous immunoglobulin replacement therapy (SCIG) when she was investigated in this study. IgE levels were elevated in patients 2 and 3 (**Table 2A**), but the clinical presentation did not indicate atopic disease.

**Table 2:** Humoral immune phenotype in TTD1 patients.

A	Serum immunoglobulins			
	TTD1 patient 1 (age: 1 year)	TTD1 patient 2 (age: 11 years)	TTD1 patient 3* (age: 18 years)	Normal range (mg/dl) (q5-q95) 1 year/11, 18 years
**IgG (mg/dl)**	135*L	857	1010	470-995/790-1700
**IgG1 (mg/dl)**	85*L	615	600	338-734/500-880
**IgG2 (mg/dl)**	55*L	198	294	41-200/150-600
**IgG3 (mg/dl)**	4*L	55	24	13-74/20-100
**IgG4 (mg/dl**	6	90	23.9	0-40/8-120
**IgM (mg/dl)**	13*L	72*L	74*L	66-161/90-350
**IgA (mg/dl)**	32	128	119	22-105/76-450
**IgE (mg/dl)**	19	404*H	767*H	<= 100
B	Serum antibody response after vaccination			
	number of vaccinations	TTD1 patient 1 (age: 1 year)	TTD1 patient 2 (age: 11 years)	Normal range (IU/ml) 1 and 11 years
**tetanus toxoid IgG (IU/ml)**	3	0.03*L	0.45	≥ 0.4
**diphtheria toxoid IgG (IU/ml)**	3	0.01*L	0.19*L	≥ 0.4
**haemophilus IgG (IU/ml)**	3	0.56*L	0.11*L	≥ 1.0
**pneumococcus IgG (titer)**	3	1:20*L	1:49*L	≥ 1:200
**pneumococcus IgM (titer)**	3	1:97*L	1:194	≥ 1:100
C	Antibody response during SCIG therapy in patient 1			
		TTD-I patient 1 (age: 13 year)		Normal range (IU/ml) (q5-q95)
**Tick-born encephalitis IgG (IU/ml)**	7	274*L		2174-11.214

Humoral immune parameters of three TTD1 patients compared to age matched control cohorts. (*Indicate examination of TTD-I patient was done during SCIG therapy, *L indicates below and *H above the normal range).

### 3.4 Naïve and transitional B-cells are decreased in peripheral blood of TTD1 patients

**Figure 5:**
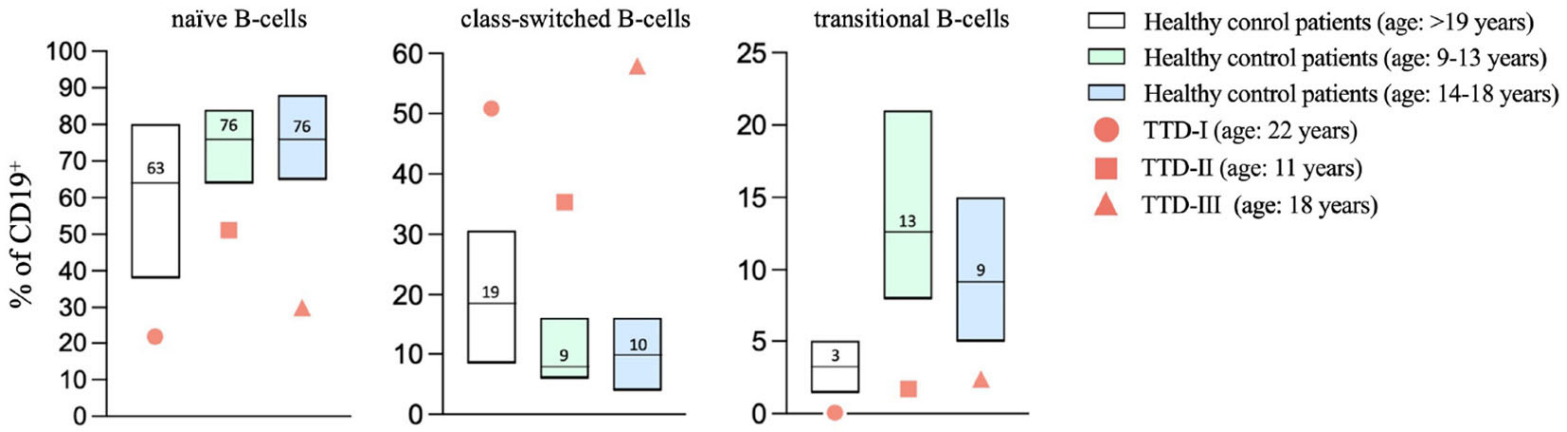
Altered B-cell maturation in TTD1. Peripheral blood samples were collected from healthy blood donors (age: ≥18 years; n=84; white bars present median, P10-P90) and *ERCC2* deficient patients (TTD-I: 22 years old, female, values shown as red bullets; TTD-II: 11 years old, male, values shown as red squares, TTD-III: 18 years old, female, values shown as red triangles). Lymphoid subpopulations were analyzed using multicolor flow cytometry. Age-matched controls for patient 2 and 3 (patient 2: age 9-13 years; n=40; light green bars represent median, P10-P90, patient 3: age 14-18 years; n=34, light blue bars represent median, P10-P90) were taken from the literature (Garcia et al., 2019). Bars represent range P10-90, line and counts indicate median values; symbols present values found in *ERCC2* deficient patients.

Investigation of B-cell subsets in peripheral blood revealed low numbers of naïve B-cells whereas class-switched B-cells were increased as compared to age matched healthy controls (**Figure 5**). Monoclonality of B-cells as a reason for higher proportions of class-switched B-cells could be excluded by repeated analyses of light-chain restriction (data not shown). Furthermore, we noticed reduced numbers of transitional B-cells.

### 3.5 Defective BCR-mediated activation of naïve B-cells in TTD1

**Figure 6:**
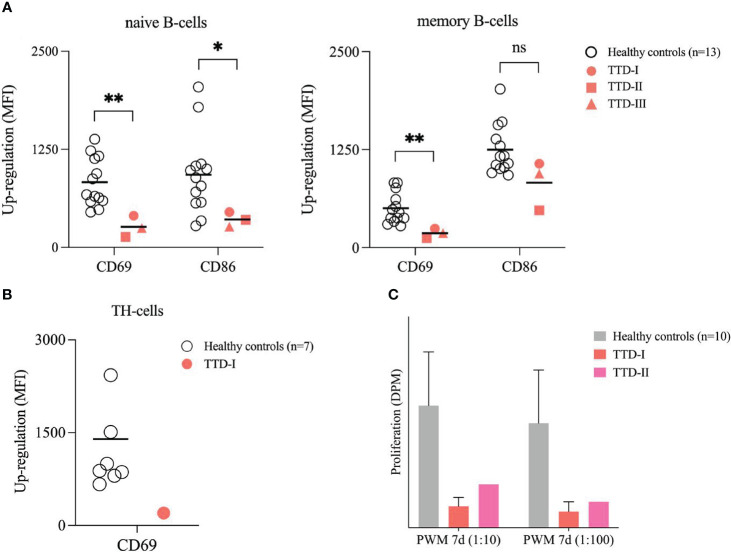
Stimulation of peripheral B- and T-cells. **(A)** Activation of B-cells in isolated PBMCs. PBMCs from 13 healthy blood donors (HCs; white bullets) and the *ERCC2* deficient patients (TTD-I: red bullet, TTD-II: red square; TTD-III: red triangle) were isolated using density gradient medium and adjusted to 1x10^6^ cells/ml medium. Cells were stimulated with SAC/IL-2 for 24 hours. Then the activation capacity was investigated using multicolor flow cytometry. CD69 and CD86 served as early activation markers on cell surface. MFI of non-stimulated cells served as reference for calculation of up-regulation. Lines indicate mean. (p-value: ns= not significant p > 0.05, *p < 0.05, **p < 0.01, non-parametric Mann-Whitney U test) **(B)** Activation of TH-cells in isolated PBMCs. PBMCs from 10 healthy blood donors (HCs; white bullets) and one *ERRC2* deficient patient 1 (TTD-I: red bullet) were isolated as described above and stimulated for 4 hours by addition of PMA/Ionomycin. The up-regulation of CD69 on the TH-cell surface was determined and calculated as described above. Lines indicate mean. **(C)** Proliferation of lymphocytes following whole blood stimulation. 10 healthy anonymous blood donors (HCs; light grey) and two TTD1 patients (TTD-I= red; TTD-II= magenta) were investigated. With medium diluted (1:20) whole blood was stimulated using different concentrations of pokeweed mitogen (PWM) and incubated for 7 days. Thymidine-[methyl-3H] was added 16-20 hours before cells were harvested for counting beta-radiation. Proliferation activity was recalculated in disintegrations per minute (DPM). Bars indicate mean values (TTD-I: 3 experiments) and error bars of HCs show standard deviation from the mean.

In healthy controls (n=13), naïve CD19^+^ B-cells up-regulated CD69 and CD86 expression 24 hours after BCR-stimulation in the presence of exogenous IL-2, whereas activation marker induction was decreased in naïve B-cells from the *ERCC2* deficient patients (**Figure 6A**). B-cell stimulation using anti-CD40 monoclonal antibodies plus exogenous IL-4 (BCR independent) did not lead to reduced B-cell activation in *ERCC2* deficient patients (data not shown). Furthermore, the stimulation of TH-cells by addition of PMA/Ionomycin to isolated PBMCs led to decreased levels of CD69 expression in the investigated patient 1 after 4 hours. (**Figure 6B**) The proliferation following whole blood stimulation using PWM (two dilutions) was decreased in lymphocytes from patient 1 and patient 2 (patient 1: three experiments, patient 3: not investigated) compared to cells from 10 healthy controls. (**Figure 6C**).

### 3.6 Expression of exponential growth response 1, 2 and 3 as well as IGH chain gene expression is significantly reduced in PBMCs of *ERCC2* deficient TTD1 patients

**Figure 7:**
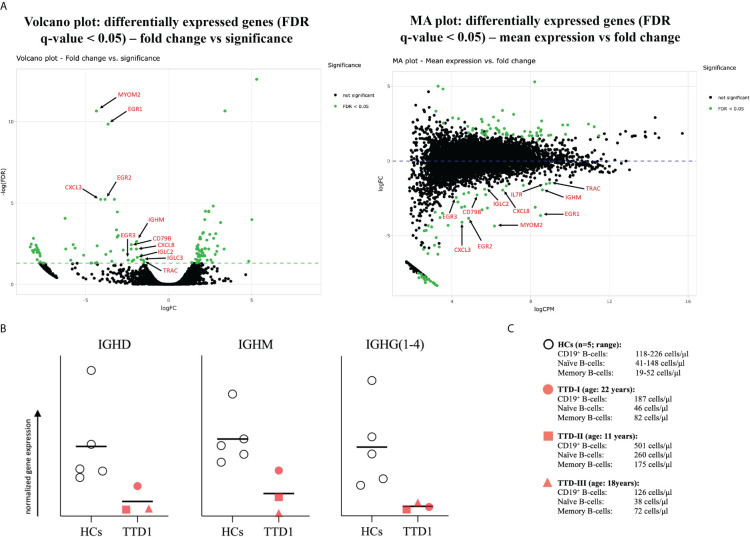
mRNA differential expression and IGH-chain analysis in PBMCs from TTD1 patients. Total RNA was isolated from PBMCs of 5 healthy controls (HCs; light grey bullets) and 3 TTD1 patients (TTD1; red symbols). Library preparation, sequencing and initial bioinformatical analysis was performed by the team of Matthias Hackl, PhD (TAmiRNA GmbH, Vienna, Austria). **(A)** mRNA differential expression analysis. Volcano and MA plots show significantly (FDR < 0.05) up- and downregulated genes (dots represent gene transcripts; green indicate significance) in PBMCs from three TTD1 patients. Transcripts of immunological importance are marked by red arrows and named by gene symbols. **(B)** Quantitative evaluation of IGH-chain gene expression. Gene expression of IGH genes (*IGHD*, *IGHM*, *IGHG*(*1* to *4*) was evaluated from mRNA sequencing data (RPM normalized) and normalized to absolute B-cell counts (*IGHD*, *IGHM* to naïve B-cell counts, *IGHG* to memory B-cells) determined in peripheral blood of each tested individual [shown in **(C)**]. Results indicate quantitative differences of IGH-chain gene expression from TTD1 patients compared to that found in HC group. Lines indicate mean values.

mRNA differential expression analysis showed that the gene expression of immunologically important activation and proliferation factors such as EGR1, EGR2, EGR3 as well as IGHM (also of light chain transcripts, data not shown) are downregulated which is in good agreement with the defective BCR-mediated B-cell activation and the antibody production observed in TTD1 patients. (**Figure 7A**) Furthermore, mRNAseq of PBMCs revealed that *IGHD*-, *IGHM*- and *IGHG*(1 to 4) gene expression normalized to peripheral blood B-cell count is reduced in the PBMCs from *ERCC2* deficient patients as compared to healthy controls. (**Figure 7B**) However, *IGHA* gene expression was not reduced in the patients (data not shown).

## 4 Discussion

In this study we report two novel *ERCC2* mutations in two unrelated patients and one previously reported *ERCC2* mutation (12) with still unknown significance in the third patient in compound heterozygous constellation together with variants already known to be pathogenic. The results presented in this study do not link the novel *ERCC2* mutations (including the previously described novel *ERCC2* mutation in the third patient) beyond doubt to the functional cell defects observed. Complementation experiments would be necessary to replicate and confirm the pathogenic effect of the new *ERCC2* mutations in a cell model. However, a number of arguments speak in favor of a pathogenic effect of the newly described mutations. We conducted in-silico prediction algorithms (PHRED, REVEL) and the results strongly predict the mutations as being pathogenic (**Table 1**). In patient 1 and 2 severely defective expression of XPD, the product of the *ERCC2* gene, could be demonstrated. The newly described *ERCC2* mutations in patient 1 (c.1378-7_1387dup) and 2 (c.494_495insT) lead to a reading frameshifts and premature stop-codons which presumably causes the loss of a large part of the XPD protein, a good explanation for the observed severely reduced XPD protein expression in this patient’s cells. It has previously been shown that the *ERCC2* missense mutation arg112his of patient 1, a common pathogenic mutation in TTD, results in severely defective protein expression (15) and that transfection with wild type XPD cDNA restores XPD protein expression in homozygote arg112his-mutated cells (15). The data presented in our study cannot explain how the *ERCC2* missense mutations present in patients 1 and 2 lead to a severely reduced protein expression. This would require a more detailed and closer consideration and experimental design in a future study, which could be initiated by our findings. Patient 3 was originally described by Chang et al. (12), and unfortunately, we were unable to test XPD protein expression in her cells. The clinical presentation of the patient (TTD, XP and immunodeficiency), the fact that her LCL cells’ UV sensitivity was defective to an extent comparable to that of cells from patients 1 and 2 strongly argue in favor of defective NER associated with *ERCC2* deficiency. For patient 1 and 2, the parents were identified as heterozygous carriers and showed no abnormalities regarding susceptibility to infections or UV sensitivity. Furthermore, no signs of TTD were observed. Additionally, we conducted immunophenotyping of the parents of patient 1 and found that neither the father (*ERCC2* c.335G>A/WT) nor the mother (*ERCC2*: c.1378-7_1387dup/WT) exhibited B cell deficiency (data not shown) or increased UV sensitivity in their LCLs (**Supplementary Figure 2**). Therefore, we assume that one-allelic *ERCC2* loss as predicted for the variant c.1378-7_1387dup does not lead to a relevant reduction of XPD-saturation in the TFIIH multiprotein complex resulting in a clinical phenotype.

Interestingly, compound heterozygous *ERCC2* null alleles (leading to loss of expression or complete dysfunction) in combination with mutations leading to impaired protein function are known to result in a more severe clinical phenotype (2). To test the pathogenicity of the *ERCC2* variants found in our patients we functionally tested proliferation and DNA repair by using the patients LCLs as a proliferative *ERCC2* deficient B-cell model. The findings of unscheduled DNA lesions characterized by increased γ-H2AX levels, increased necrotic cell death, and decreased proliferation in the *ERCC2* deficient patients following UV-irradiation confirmed impaired NER in all three patients. Additionally, we could confirm these findings in PBMCs of one TTD1 patient and could detect defective NER in peripheral B-cells. In contrast, the response to neocarzinostatin treatment was normal showing *ERCC2* deficiency does not result in the early DNA double strand break (DSB) response defect as found in AT- or NBS patients (shown in **Supplementary Figure 1**).

An important contribution to cellular viability and function mediated by XPD apart from maintenance of the genomic stability is the regulation of metabolism and cell growth by involvement in gene expression (16, 17). XPD acts as a 5’ - 3’ helicase subunit in the TFIIH transcription complex for the opening of the DNA double strand around promotor regions, a process finally needed for protein synthesis following gene expression of class II genes (2, 6). Thus, it is not surprising that TTD is considered a transcription syndrome besides a NER defect (2, 18). Impaired transcription in physiologically high expressed genes has been demonstrated by reduced human hemoglobin gene expression in TTD patients (2, 18) suggesting that up-regulation of transcription may be impaired in TTD due to dysfunctions and/or quantitative reduction of XPD. A quantitative reduction of XPD protein as found in LCLs of our TTD1 patients explains the reduced efficiency of class II gene transcription as well as DNA repair. These pathways are essential for B-cell differentiation and maturation to active antibody producing plasma cells (19). Furthermore, chemical stress such as the accumulation of reactive oxygen species (ROS) during cell proliferation can cause DNA damage (20) that could have harmful effects in activated TTD1 B-cells if not processed effectively.

In *ERCC2*-deficiency, other NER genes partially compensate for the loss of XPD, thus explaining the milder clinical phenotype of TTD1 as compared to xeroderma pigmentosum (XP) with respect to defective DNA repair (2). A defect in other NER factors (without involvement in transcription process) leads to the photosensitive disease XP. Thus trichothiodystrophy (TTD) can be seen as a transcription syndrome in addition to a defect in DNA repair (2). The fact that defective tRNA synthetases (MARS1 and AARS1) can lead to non-photosensitive TTD supports the hypothesis that the clinical phenotype of TTD is more likely due to impaired general transcription (or translation) (21). This indicates that the immunological phenotype found in B cells is also more likely due to a transcription defect than solely NER dysfunction. XPD participates in the multiprotein complex TFIIH making it unlikely that other NER factors could ameliorate the helicase dysfunction in transcription of important B cell genes (especially transcription initiation and transcription coupled DNA repair). In this study we investigated LCL cells from two NER knockout patients suffering from Xeroderma pigmentosum (XPA -/- (homozygous: *XPA*: NM_000380.4:c.368del, NP_000371.1: p.Leu123fs) and (XPF -/- (*ERCC4*: NM_0052236.3:c1811+1G>A)/NM_005236.3: c.2395C>T, NP_005227.1: p.Arg799Trp)) as positive controls for establishing maximum UV sensitivity. However, these XP patients showed no altered B-cell subsets as found in the TTD1 patients (data not shown), thus indicating that other NER factors are unlikely to compensate for the B cell abnormalities described in *ERCC2*-deficiency, and rather points to the unique role *ERCC2* might play in regulating transcription of essential B cell factors as an explanation for the immunodeficient phenotype.

Along these lines our results show impaired antibody responses *in-vivo* which have rarely been demonstrated in TTD1 patients before (12). Interestingly, Serum IgG levels were normal in patient 2 and – in contrast to patients 1 and 3, this patient did not receive immunoglobulin replacement therapy. However, IgG response to vaccination was impaired in this patient. The patient presented a history of recurrent infection since the early years of life. It would be possible that in the first years of life, hypogammaglobulinemia was present in patient 2, at a time when he was not presented to immunological work-up. Further investigation of very young *ERCC2*-deficient patients would be needed to show changes in serum-IgG levels over time. In this context it is important to note that B-cell deficiency is present in more than 70% of PID patients (22) and that several inborn errors of immunity can lead to antibody deficiency (23). Our results should encourage the immunological investigation of patients harboring *ERCC2* mutations, as well as mutation analysis of *ERCC2* in patients with primary antibody deficiency with unknown molecular mechanism. Even though DNA repair deficiency is known to lead to immunodeficiency (19, 24), it is notable that in the newest update of the International Union of Immunological Societies (IUIS) Expert Committee in 2022, most of the listed DNA repair genes are involved in DSB repair (25) while defects in NER-associated genes such as *ERCC2* are not included and relatively few proteins involved in NER are yet associated with immunodeficiency (3, 24). In this context our study shows that *ERCC2* might be a likely candidate to be included in the list of NER-associated gene mutations causing antibody deficiency.

Furthermore, we could demonstrate that TTD1 patients presented with decreased transitional B-cells and naïve B-cells associated with a compensatory relative increase in memory B-cells. Decreased numbers of naïve B-cells are known to be associated with impaired antibody response and hypogammaglobulinemia (26) leading to severe infections and defective or missing transitional B-cell subsets are described as being associated with immune-dysregulation and immunodeficiency (27). Our findings could indicate that *ERCC2* deficiency can affect B-cell maturation in the bone marrow leading to decreased replenishment of appropriate numbers of transitional and naïve B-cells to peripheral blood and probably lymphoid tissue. However, the investigation of bone marrow and lymphoid tissues or kappa receptor excision circle (KREC) analysis of several TTD1 patients would be needed to support this hypothesis. We didn’t have the opportunity to investigate the bone marrow of our patients, and our findings should stimulate future studies to clarify this point. An alternative explanation for the reduced percentages of naïve and transitional B-cells found in peripheral blood of TTD1 patients might be that impaired DNA repair and transcription initiates premature aging events in TTD1 B-cells leading to abnormal distribution of the peripheral B-cell subsets. This phenomenon has been found in other DNA repair disorders (28, 29) and has been partially described in TTD mice (30, 31). In AT patients it has been reported that T- and B-cell subsets present with disturbed aging profiles as compared to healthy patients characterized by reduced naïve T- and B-cells but relatively increased memory T- and B-cells (32). Therefore, our findings underline the importance of DNA repair during the development and replenishment of naïve lymphocytes as shown in other DNA repair defects.

We found reduced BCR-mediated B-cell activation in naïve B-cells of TTD1 patients indicating that the XPD protein is important for early B-cell activation. BCR stimulation and the resulting early activation events are highly needed during maturation of naïve and transitional B-cells (26, 27, 33). CD69 and CD86 represent early cell activation markers and have several functions in immune regulation as well as B-cell activation (34, 35). Reduced expression of these activation markers following BCR stimulation in naïve and memory B-cells of the TTD1 patients indicate that functional XPD is needed during early B-cell activation. Impaired BCR mediated B-cell activation likely contributes to the antibody deficiency in TTD1 deficient patients, however it is not excluded that the previously reported defects in inborn immunity and T-cell functions might also contribute to impaired antibody production in *ERCC2* deficiency. Along these lines we investigated T-cell activation in patient 1 and found reduced expression of CD69 on PMA+IM-stimulated TH cells. This in good agreement with the defects in T-cells and dendritic cells described in the literature (8, 9), and together with the impairment in early B-cell activation is likely to contribute to the antibody deficiency. In this context, it is noteworthy that we found reduced lymphocyte proliferation responses following pokeweed mitogen (PWM) stimulation of peripheral blood lymphocytes, known to involve both T- and B-cell stimulation, in the two TTD1 patients tested (**Figure 6C**).

Our finding of reduced IGH transcript levels as well as downregulated genes such as *EGR 1*, *2* and *3* indicate that impaired immunoglobulin gene transcription might contribute to the defect in B-cell function found in our patients. These growth factors are highly needed for the activation of T- and B-cells and the response to mitogens (36, 37). EGR1 is upregulated in B cells following stimulation through BCR crosslinking but not in response to anti-CD40 stimulus in mice. Additionally, knockout of EGR1 resulted in defective immune responses following stimulation via BCR crosslinking (35). With our results, we were able to demonstrate downregulated *EGR1* transcripts in the PBMCs of *ERCC2*-deficient patients. Furthermore, we functionally demonstrated a reduced activation capacity in naïve B-cells following BCR-mediated B-cell activation, which was not observed with BCR-independent stimulation. This indicates the importance of XPD function in BCR activation pathways. However, EGR2 and EGR3 were also identified as having important functions as early regulators of immune response, directly involved in both B-cell and T-cell activation (38, 39). A quantitative lack of EGR2 could contribute to the reduced immune response often found in trichothiodystrophy patients. Total IgM was the only serum immunoglobulin which was decreased in all TTD1 patients in this study, and the differential expression analysis confirmed that *IGHM* gene expression was significantly downregulated in our patients. The comparison of *IGHD*, *IGHM* and *IGHG*(1 to 4) gene expression in PBMCs from TTD1 patients and healthy controls showed reduced gene expression of IGH chain genes in the patients thus indicating transcriptional impairment in B-cells likely to result in antibody deficiency. An additional finding was that several of the genes expressed in healthy control B-cells at a high level represented ribosomal proteins which were reduced at the transcriptional level in TTD1 cells (data not shown). In this context it is interesting that recently dysfunctional biogenesis of ribosomes was reported as a novel pathomechanism in TTD (40). Based on our findings we propose that quantitatively reduced gene expression of house-keeping factors, EGR factors as well as IGH chain genes contribute to impaired B-cell function in TTD1 patients. Reduced expression of these genes might be particularly relevant for impaired immune cell function in TTD1 when these cells are in an active and proliferating state during an immune response.

Taken together our findings revealed impaired BCR-mediated B-cell activation and transcriptional downregulation of important growth factors, leading to impairment of humoral immunity. The present results indicate that patients with confirmed *ERCC2* deficiency or the diagnosis of TTD1 should be investigated for antibody deficiency to enable early initiation of antibody replacement therapy, which could protect *ERCC2* deficient patients from acquiring severe and potentially life-threatening infections.

## Data availability statement

The NGS datasets presented in this study can be found in online repositories. The names of the repository/repositories and accession number(s) can be found below: https://www.ncbi.nlm.nih.gov/, GSE262217.

## Ethics statement

The study was conducted in accordance with the Declaration of Helsinki and the guidelines of the Austrian Agency of Research Integrity (OeAWI). With respect to the clinical immunological analyses this study was approved by the Ethics Committee of the Immunology Outpatient Clinic as a study using the residual specimen’s biobank of the Immunology Outpatient Clinic. According to the Ethics Committee of the City of Vienna and the legal regulations to be applied (§15a Abs. 3a Wiener Krankenanstaltengesetz) no additional ethics committee evaluation is required for a non-interventional study using data collected as part of the routine medical care the patients received. The families of the patients gave their informed consent that anonymized data collected as part of the routine medical attendance (serum antibody and flow cytometry analysis, activation-induced marker assay) could be included in a scientific publication. All patient information in this study is anonymized and deidentified. No extra intervention was carried out.

## Author contributions

RR: Conceptualization, Data curation, Formal analysis, Investigation, Methodology, Validation, Visualization, Writing – original draft, Writing – review & editing. KS: Methodology, Writing – review & editing. CG: Supervision, Validation, Writing – review & editing. AL-P: Data curation, Methodology, Writing – review & editing. RS: Methodology, Writing – review & editing. SS: Resources, Writing – review & editing. RG: Resources, Writing – review & editing. HB: Supervision, Writing – review & editing. JV: Resources, Writing – review & editing. KC: Resources, Writing – review & editing. JW: Resources, Supervision, Writing – review & editing. HW: Conceptualization, Formal analysis, Investigation, Supervision, Validation, Writing – original draft, Writing – review & editing.
