# Supplementary material for: Impaired B-cell function in ERCC2 deficiency
Source: Front Immunol. 2024 Jul 11;15:1423141. doi: 10.3389/fimmu.2024.1423141 (PMC11269123; doi:10.3389/fimmu.2024.1423141)
Supplement: Supplementary file 1 [file Presentation_1.pptx]

## Slide 1
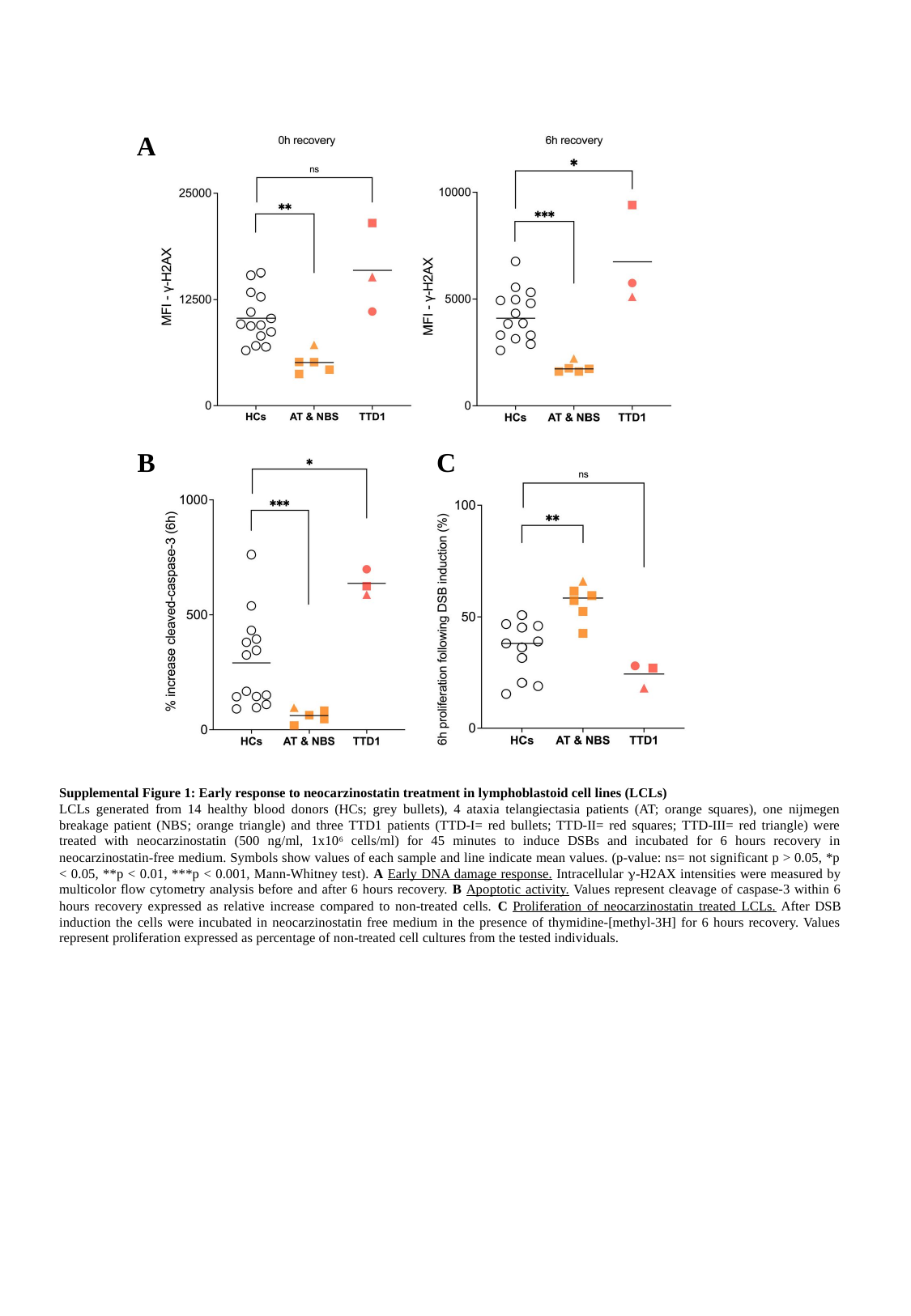

A
B
C
Supplemental Figure 1: Early response to neocarzinostatin treatment in lymphoblastoid cell lines (LCLs)
LCLs generated from 14 healthy blood donors (HCs; grey bullets), 4 ataxia telangiectasia patients (AT; orange squares), one nijmegen breakage patient (NBS; orange triangle) and three TTD1 patients (TTD-I= red bullets; TTD-II= red squares; TTD-III= red triangle) were treated with neocarzinostatin (500 ng/ml, 1x10^6^ cells/ml) for 45 minutes to induce DSBs and incubated for 6 hours recovery in neocarzinostatin-free medium. Symbols show values of each sample and line indicate mean values. (p-value: ns= not significant p > 0.05, *p < 0.05, **p < 0.01, ***p < 0.001, Mann-Whitney test). A Early DNA damage response. Intracellular γ-H2AX intensities were measured by multicolor flow cytometry analysis before and after 6 hours recovery. B Apoptotic activity. Values represent cleavage of caspase-3 within 6 hours recovery expressed as relative increase compared to non-treated cells. C Proliferation of neocarzinostatin treated LCLs. After DSB induction the cells were incubated in neocarzinostatin free medium in the presence of thymidine-[methyl-3H] for 6 hours recovery. Values represent proliferation expressed as percentage of non-treated cell cultures from the tested individuals.

## Slide 2
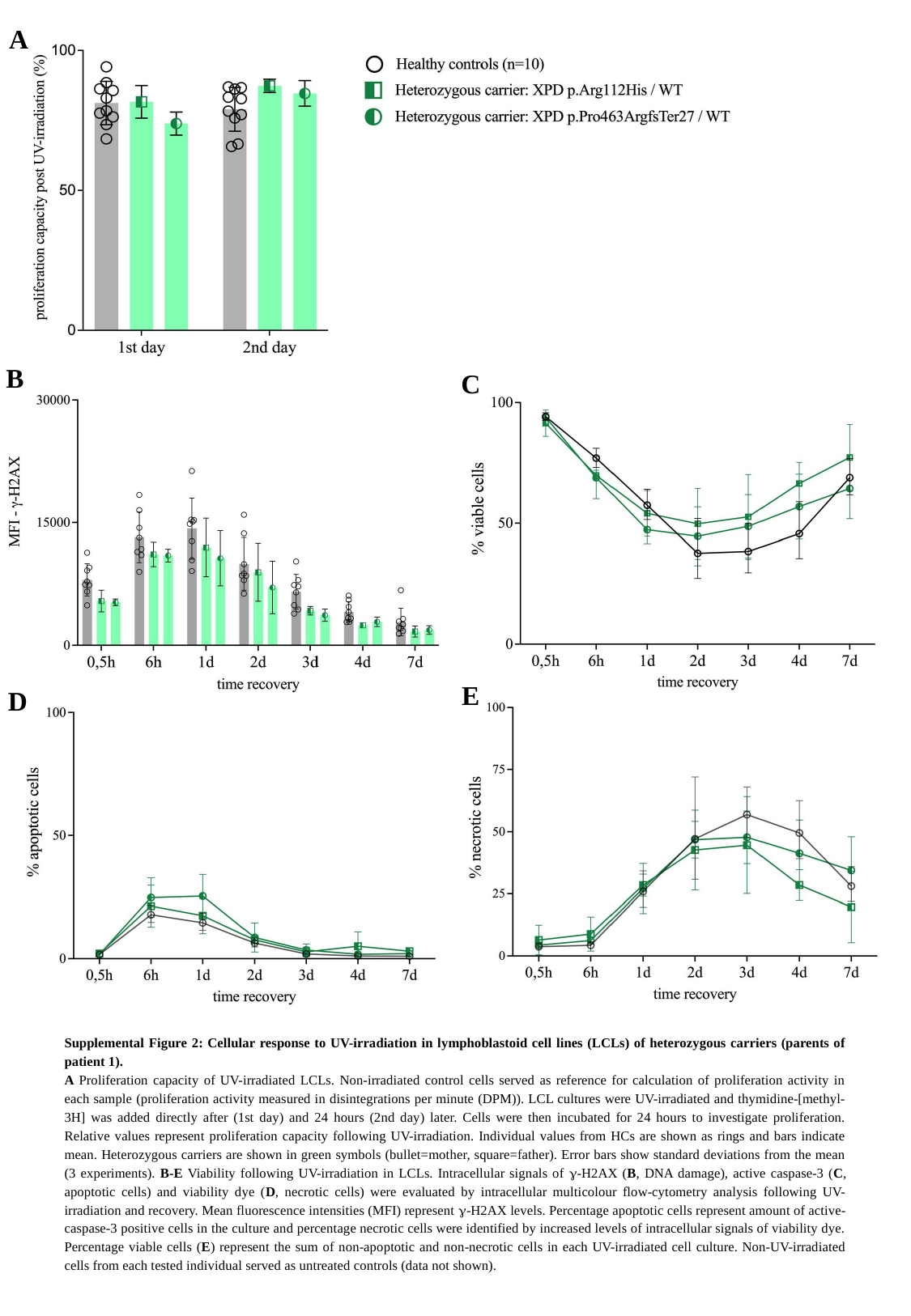

A
B
C
E
D
Supplemental Figure 2: Cellular response to UV-irradiation in lymphoblastoid cell lines (LCLs) of heterozygous carriers (parents of patient 1).
A Proliferation capacity of UV-irradiated LCLs. Non-irradiated control cells served as reference for calculation of proliferation activity in each sample (proliferation activity measured in disintegrations per minute (DPM)). LCL cultures were UV-irradiated and thymidine-[methyl-3H] was added directly after (1st day) and 24 hours (2nd day) later. Cells were then incubated for 24 hours to investigate proliferation. Relative values represent proliferation capacity following UV-irradiation. Individual values from HCs are shown as rings and bars indicate mean. Heterozygous carriers are shown in green symbols (bullet=mother, square=father). Error bars show standard deviations from the mean (3 experiments). B-E Viability following UV-irradiation in LCLs. Intracellular signals of γ-H2AX (B, DNA damage), active caspase-3 (C, apoptotic cells) and viability dye (D, necrotic cells) were evaluated by intracellular multicolour flow-cytometry analysis following UV-irradiation and recovery. Mean fluorescence intensities (MFI) represent γ-H2AX levels. Percentage apoptotic cells represent amount of active-caspase-3 positive cells in the culture and percentage necrotic cells were identified by increased levels of intracellular signals of viability dye. Percentage viable cells (E) represent the sum of non-apoptotic and non-necrotic cells in each UV-irradiated cell culture. Non-UV-irradiated cells from each tested individual served as untreated controls (data not shown).
